# Supplementary material for: A conserved inhibitory interdomain interaction regulates DNA-binding activities of hybrid two-component systems in Bacteroides
Source: mBio. 2024 Jun 6;15(7):e01220-24. doi: 10.1128/mbio.01220-24 (PMC11253607; doi:10.1128/mbio.01220-24)
Supplement: Supplemental Materials — Supplemental text, figures, and tables. [file mbio.01220-24-s0001.pdf]

Supplemental Materials

Supplemental Figure S1-S8

Supplemental Table S1-S3

Supplemental Text

A Conserved Inhibitory Interdomain Interaction Regulates DNA-binding Activities of Hybrid Two-component Systems in *Bacteroides*

Rong Gao, Ti Wu, and Ann M. Stock*

Center for Advanced Biotechnology and Medicine, Department of Biochemistry and Molecular Biology, Rutgers University - Robert Wood Johnson Medical School, Piscataway, New Jersey 08854, USA

Supplemental Fig. S1

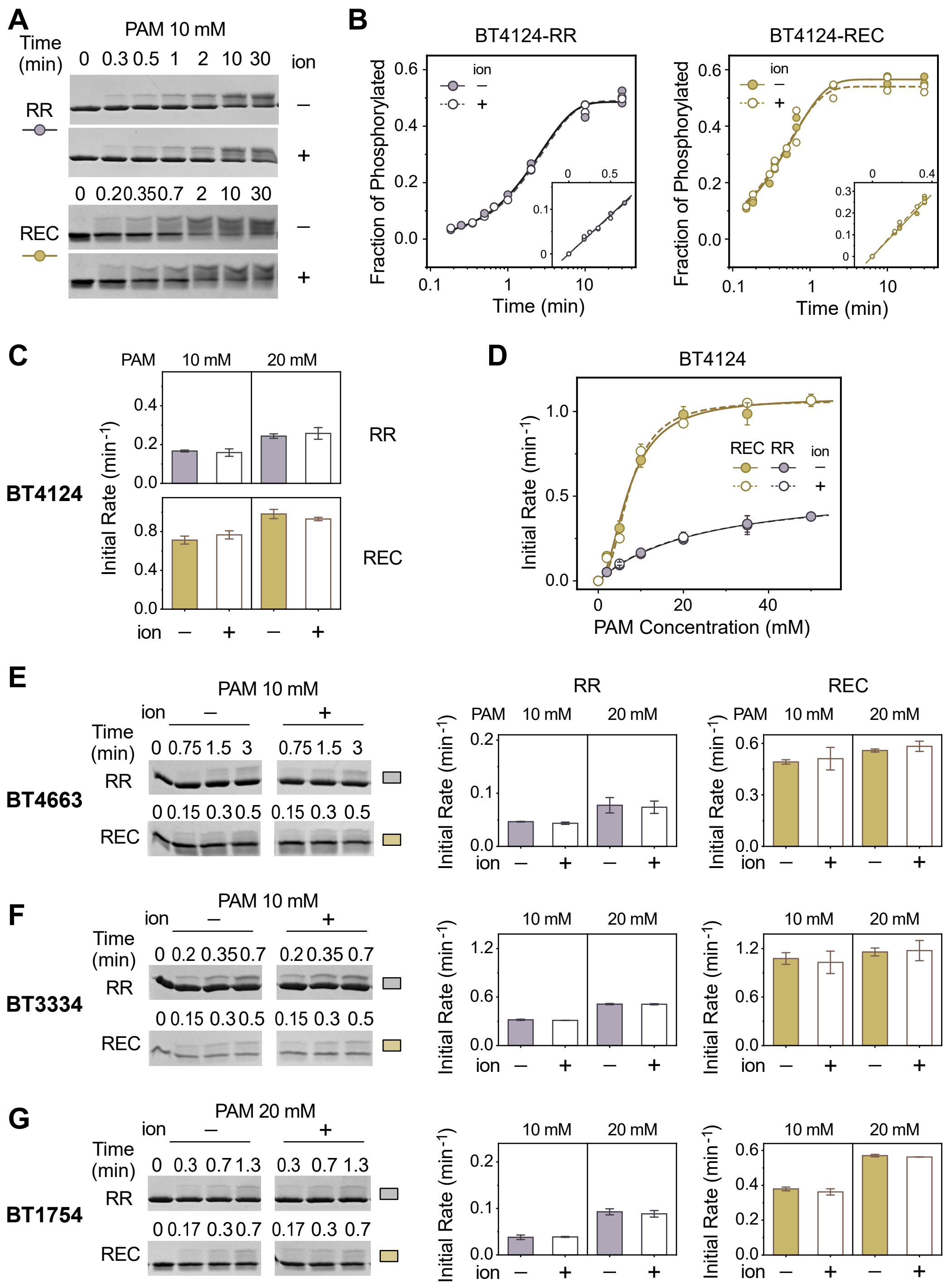

FIG S1. Autophosphorylation of HTCS proteins is not significantly affected by ionic strength. Phosphorylation kinetics of BT4124 (A-D), BT4663 (E), BT3334 (F) and BT1754 (G) with no salt addition (-) or with added NaCl to reach a constant ionic strength at 0.22 M (+). One representative example of Phos-tag gels at the indicated PAM concentration is shown for each protein. Quantification of Phos-tag gels yielded the phosphorylation time course (B) and the early stage of the time course (B inset) was used to calculate the initial rates (C, D, mid and right panels in E, F and G).

Supplemental Fig. S2

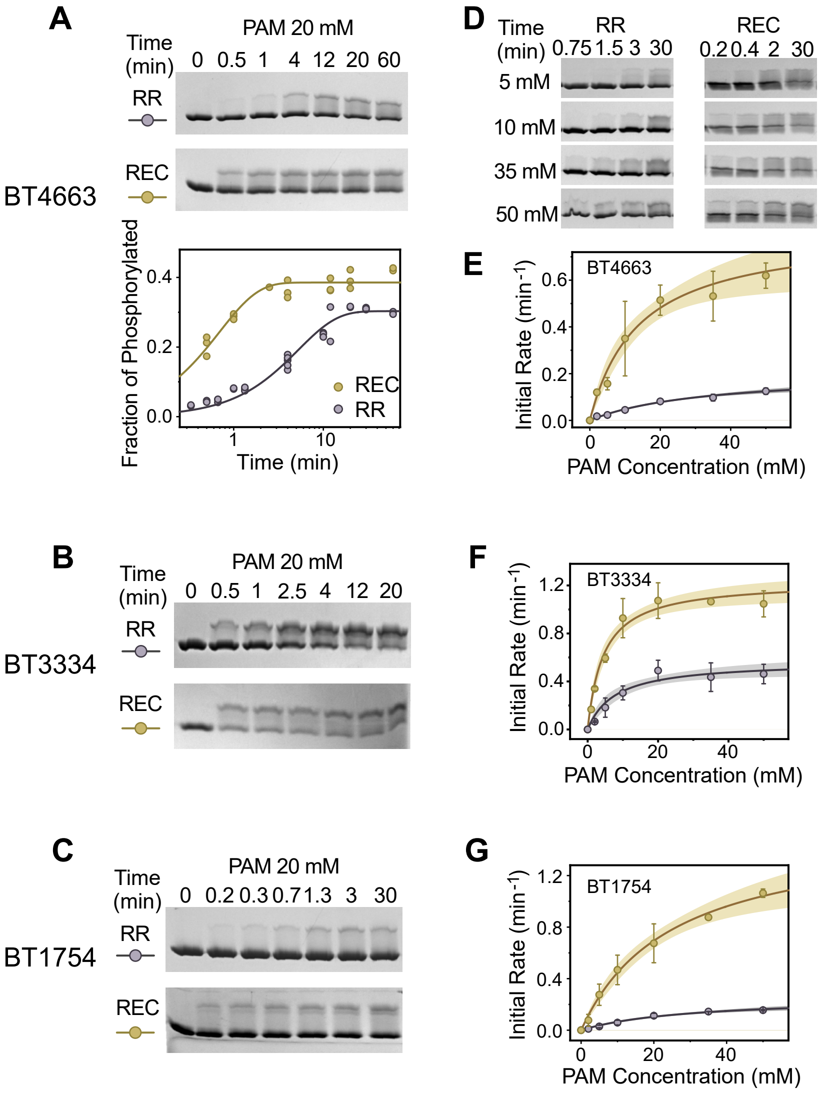

FIG S2. Autophosphorylation kinetics of HTCS-RR and HTCS-REC proteins. (A-C) Phos-tag gel analyses of protein phosphorylation at 20 mM PAM for BT4663, BT3334 and BT1754. One representative example is shown for each protein. The lower panel of (A) shows an example of phosphorylation time course of BT4663 based on Phos-tag gel quantification. (D) Phosphorylation of BT4663 at different PAM concentrations. One representative example is shown for each condition. Longer times were used for BT4663-RR to allow sufficient phosphorylation to be observed and quantified for calculating the initial rates. (E-G) Dependence of phosphorylation initial rates on PAM concentrations. Data are shown as mean ± SD from at least three independent experiments. Lines with corresponding shaded ranges indicate the fitted curves and the 95% confidence intervals. The shaded ranges for BT4633-RR and BT1754-RR are barely noticeable due to a narrow confidence interval.

Supplemental Fig. S3

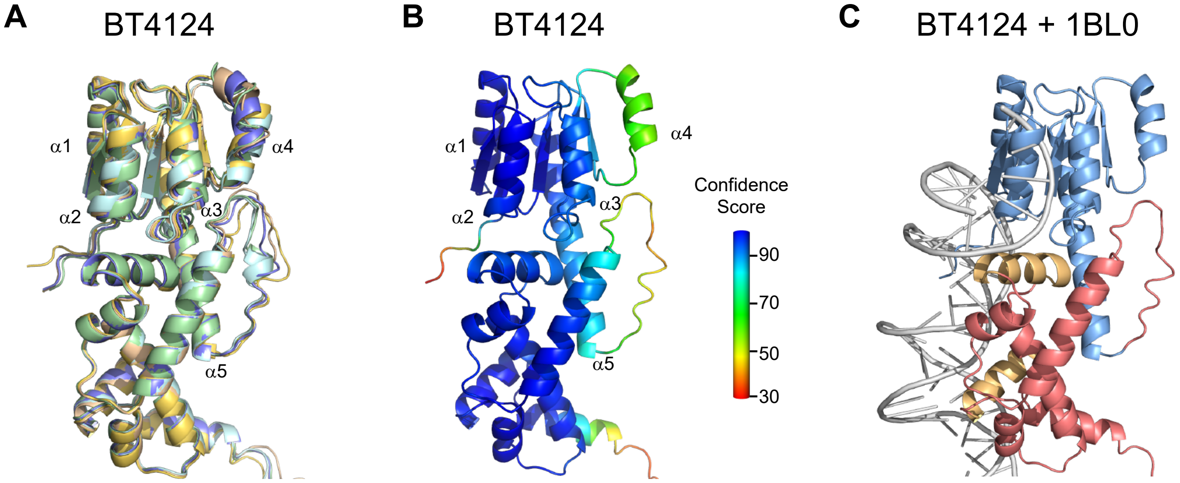

FIG S3. Structures of HTCS-RRs predicted by AlphaFold 2. (A) Superposition of the five top-ranked BT4124-RR structures. From rank 1 to rank 5, structures are colored violet, light cyan, green, yellow and wheat. All five structures superimpose well with each other, except for helix α4 and the connecting loop (α5-α6) between the REC and DBD domains. (B) BT4124 structure colored with the pLDDT confidence score. The majority of the structure, except for the α5-α6 connecting loop, the N- and C-termini, have high confidence scores above 50. (C) Superposition of the predicted BT4124-RR structure with the DNA-bound HTH18 family member, MarA (1BL0). The domain arrangement in the predicted BT4124-RR structure appears to not allow DNA-binding due to the steric clash of the REC domain with DNA.

Supplemental Fig. S4

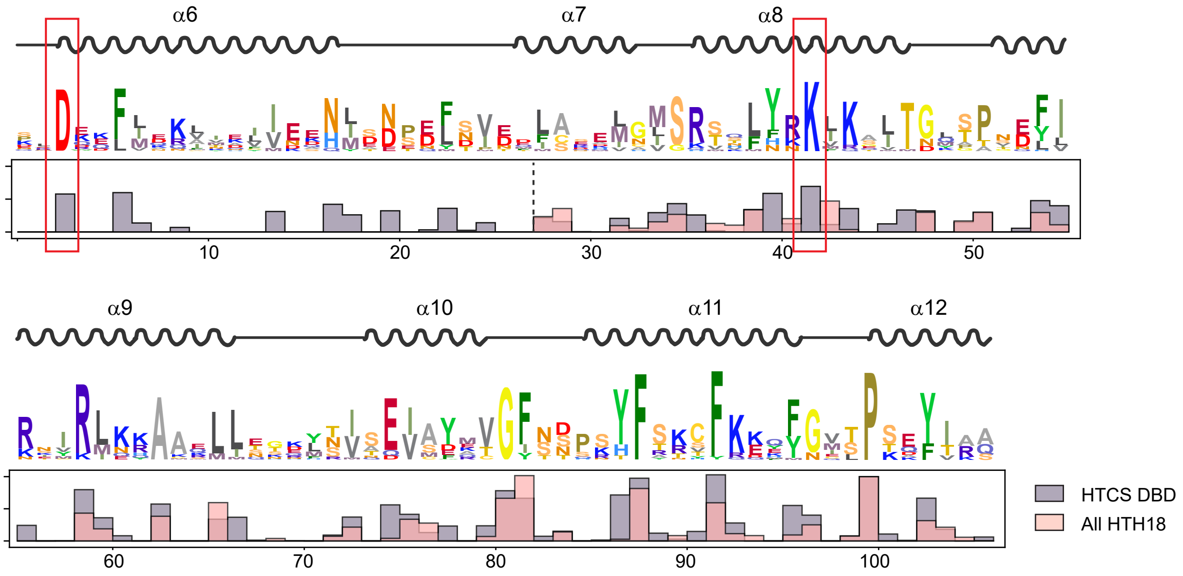

FIG S4. Sequence conservation of HTCS-DBD domains. Sequence logos were obtained from the profile hidden Markov model of 6908 HTCS proteins in *Bacteroides*. Secondary structural elements are shown above the logos. DBD domains of HTCSs belong to the HTH18 family (PFAM id, PF12833). Bar graphs compare the normalized information contents (ICs) between HTCS-DBDs (gray) and the entire HTH18 family (pink). HMM of HTH18 only contains residues starting from helix α7 (vertical dashed line), thus ICs were not calculated for HTH18 preceding α7. Residues involved in the predicted REC-DBD interfaces (red box) appear highly conserved in HTCS-DBDs, but not in the entire HTH18 family.

Supplemental Fig. S5

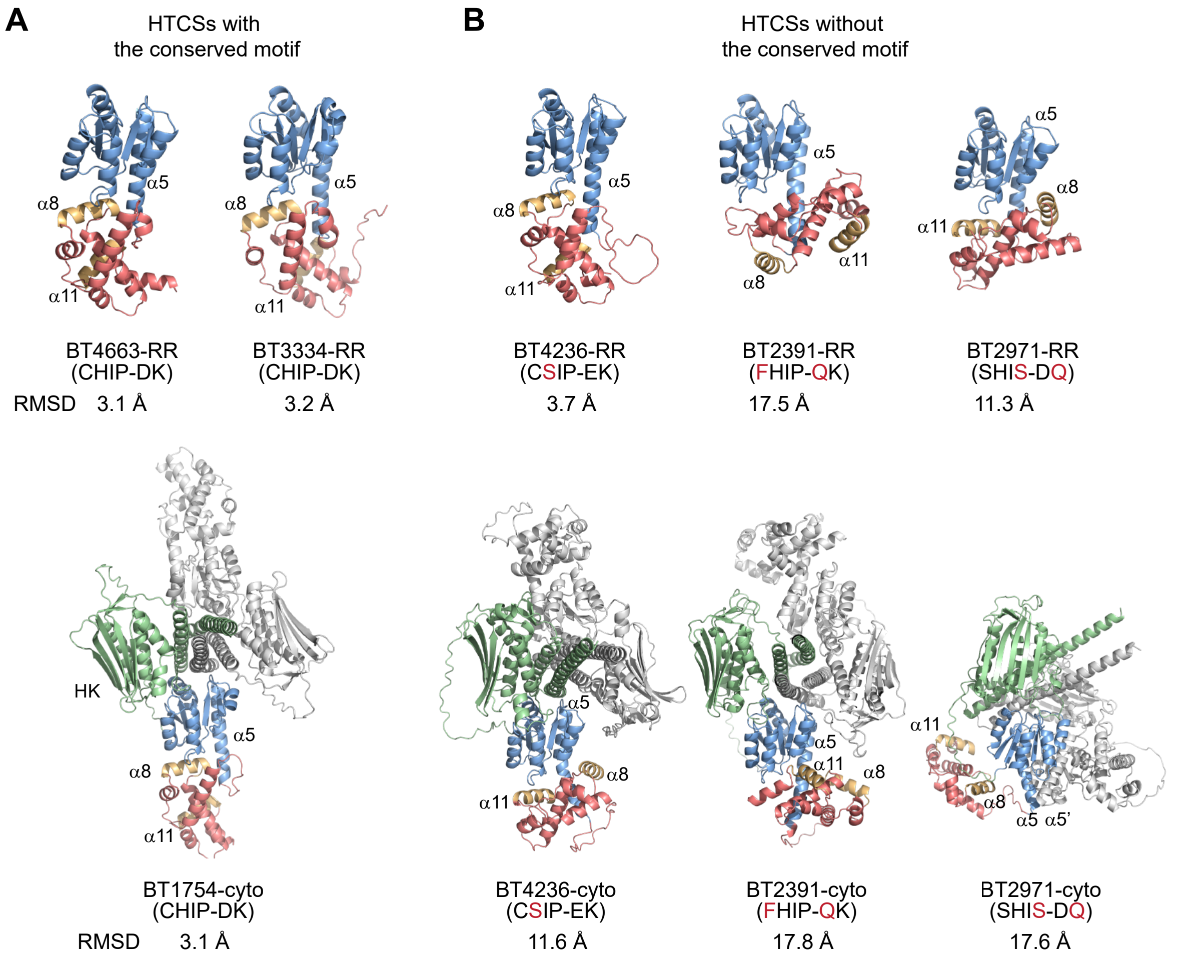

FIG S5. Predicted structures of HTCSs from *B. theta*. RMSD values are derived from structural alignment with BT4124-RR. (A) Similar REC-DBD domain orientation for HTCSs containing the conserved S/CHIP-DK motif. Small RMSD values indicate high structural similarity to BT4124. For BT1754, the conserved REC-DBD domain orientation is only observed in the predicted dimeric structure of BT1754-cyto that contains both the HK and RR domains. (B) Diverse domain orientations in HTCSs that have motif sequences that deviate from the consensus. The helix α5 in the REC domain, the DNA recognition helices α8 and α11 are labeled to highlight different orientations of the DBD. All structures shown are the top ranked predictions from AlphaFold. For HTCSs without the consensus motif, low ranked predictions (rank 2-5) also have large RMSD values, suggesting REC-DBD orientations distinct from the conserved one observed in BT4124-RR.

Supplemental Fig. S6

BT2628-RR

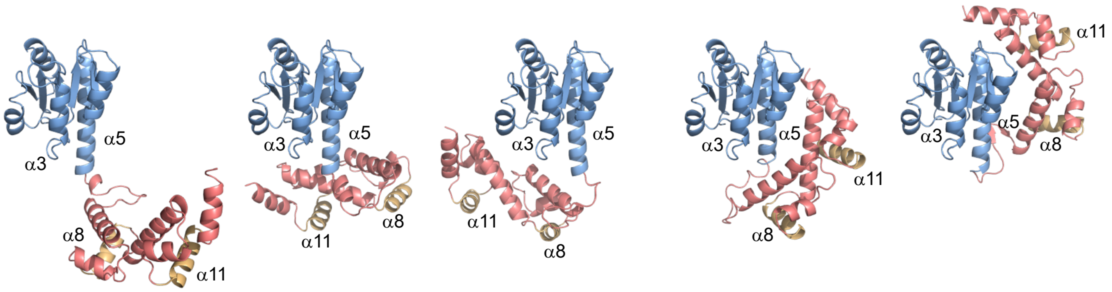

	Rank 1	Rank 2	Rank 3	Rank 4	Rank 5
pLDDT	87.3	86.8	86.1	82.4	82.2
pTM	0.543	0.535	0.526	0.514	0.506

FIG S6. Plasticity of domain orientations for different ranked structural predictions. One representative, BT2628-RR, is shown here with five different DBD orientations for five ranked predictions. All ranked predictions have high pLDDT scores, suggesting well-modelled local structural elements. The pTM scores are at intermediate values of ~0.5 not dramatically different for five ranked predictions, suggesting the inter-domain accuracy is not high, likely caused by the relative mobility of individual domains.

Supplemental Fig. S7

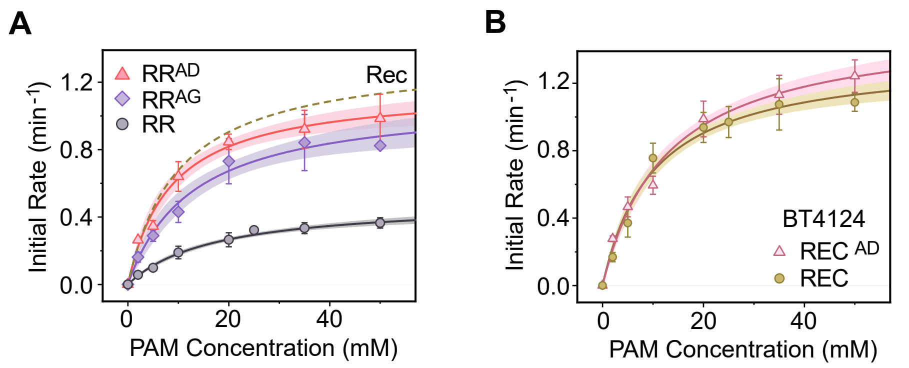

FIG S7. Autophosphorylation kinetics of BT4124 domains and corresponding interface variants. Initial rates of phosphorylation at different PAM concentrations were measured from phos-tag gels shown in Figure 5. Lines with corresponding shaded ranges indicate the curves fitted with the Michaelis-Menten equation and the 95% confidence intervals. (A) Differences in phosphorylation kinetics are apparent for BT4124-RR and corresponding interface variants, BT4124-RR^{AG} and BT4124-RR^{AD}, suggesting differences in the inhibitory interactions between the REC and DBD domains. (B) BT4124-REC and BT4124-REC^{AD} have similar rate curves with overlapping confidence ranges, suggesting that the substitution in the isolated receiver domain did not alter the phosphorylation efficiency.

Supplemental Fig. S8

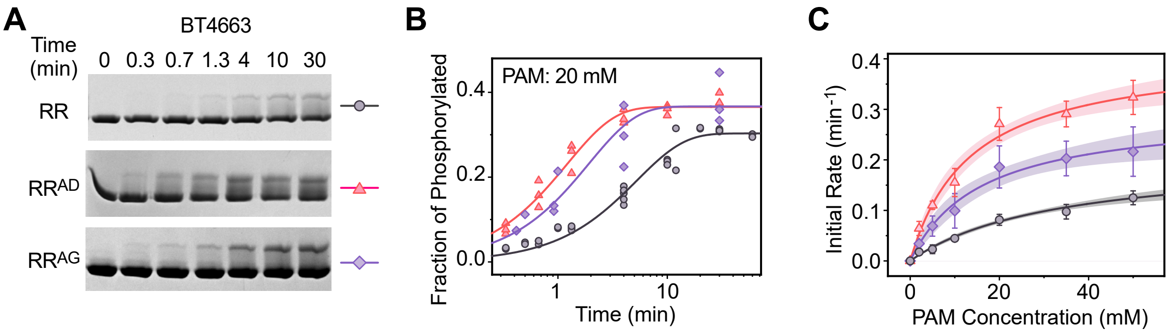

FIG S8. Autophosphorylation kinetics of BT4663-RR and corresponding interface variants. Phos-tag gels (A) were quantified to track the fraction of phosphorylated proteins (B) at indicated times after addition of 20 mM PAM. Lines in (B) represent the global exponential fit to illustrate the kinetic trendline of phosphorylation. Initial rates of phosphorylation were measured from early stages of the reaction to derive the kinetic curves in (C). Similar to that observed for BT4124, the interface variants, BT4663-RR^{AG} and BT4663-RR^{AD}, showed faster phosphorylation kinetics than BT4663-RR, suggesting relief of inhibitory interactions.

Supplemental Tables

Table S1. Phosphorylation efficiency (k_{cat}/K_m) for autophosphorylation of HTCS RR proteins.

Proteins	Relative efficiency	Phosphorylation efficiency ($min^{-1}mM^{-1}$) [a]	k_{cat} (min^{-1}) [a]	K_m (mM) [a]	# of data points [b]
cooperativity h =1, Michaelis-Menten kinetics					
BT4124-Rec	1	0.14 ± 0.02	1.4 ± 0.1	10 ± 1	44
BT4124-RR	0.21	0.03 ± 0.004	0.50 ± 0.03	17 ± 2	39
BT4124-RR^{AG}	0.64	0.09 ± 0.02	1.1 ± 0.1	13 ± 3	22
BT4124-RR^{AD}	1.0	0.14 ± 0.03	1.2 ± 0.1	8.5 ± 1.5	22
BT4124-Rec^{AD}	0.91	0.12 ± 0.02	1.6 ± 0.1	13 ± 2	29
BT4663-Rec	1	0.06 ± 0.02	0.81 ± 0.1	14 ± 4	28
BT4663-RR	0.11	0.006 ± 0.001	0.21 ± 0.02	33 ± 6	35
BT4663-RR^{AG}	0.32	0.02 ± 0.01	0.29 ± 0.03	16 ± 5	22
BT4663-RR^{AD}	0.52	0.03 ± 0.01	0.41 ± 0.03	14 ± 3	20
BT3334-Rec	1	0.28 ± 0.05	1.2 ± 0.1	4.5 ± 0.8	26
BT3334-RR	0.26	0.07 ± 0.02	0.57 ± 0.05	7.8 ± 2.4	24
BT1754-Rec	1	0.06 ± 0.02	1.6 ± 0.2	25 ± 6	18
BT1754-RR	0.14	0.009 ± 0.002	0.26 ± 0.03	30 ± 7	24

a. Values represent the fitted parameter values with standard error.
b. The number of data points indicates the number of initial rates used for the fitting to derive k_{cat} and K_m. Initial rates were measured at different PAM concentrations from multiple independent experiments.

Table S2. Strains and plasmids used in this study.

Strains / plasmids	Relevant characteristics	Reference / source
Strains *E. coli*		
DH5α	General cloning strain	Invitrogen
BL21(DE3)	F^{-}, *omp*T, *hsd*S_B(r_B-, m_B-), *dcm*, *gal*, λ(DE3)	Novagen
Plasmids		
pT7GG2	Golden gate cloning vector for HTCS expression, P_{T7}-MCS-6xHis, *lacI*^{q}, Ap^r	This study
pT7-bt1754-RR	BT1754-RR (BT1754 aa670 - end), Ap^r	This study
pT7-bt1754-Rec	BT1754-Rec (BT1754 aa670 - 804), Ap^r	This study
pT7-bt3334-RR	BT3334-RR (BT3334 aa1089 - end), Ap^r	This study
pT7-bt3334-Rec	BT3334-Rec (BT3334 aa1089 - 1225), Ap^r	This study
pT7-bt4124-RR	BT4124-RR (BT4124 aa1186 - end), Ap^r	This study
pT7-bt4124-Rec	BT4124-Rec (BT4124 aa1186 - 1323), Ap^r	This study
pT7-bt4124-RR^{AD}	BT4124-RR^{AD} (BT4124 aa1186 - end, C1262A/H1263D), Ap^r	This study
pT7-bt4124-Rec^{AD}	BT4124-Rec^{AD} (BT4124 aa1186 - 1323, C1262A/H1263D), Ap^r	This study
pT7-bt4124-RR^{AG}	BT4124-RR^{AG} (BT4124 aa1186 - end, C1262A/H1263G), Ap^r	This study
pT7-bt4663-RR	BT4663-RR (BT4663 aa1097 - end), Ap^r	This study
pT7-bt4663-Rec	BT4663-Rec (BT4663 aa1097 - 1232), Ap^r	This study
pT7-bt4663-RR^{AD}	BT4663-RR^{AD} (BT4663 aa1097 - end, C1174A/H1175D), Ap^r	This study
pT7-bt4663-RR^{AG}	BT4663-RR^{AG} (BT4663 aa1097 - end, C1174A/H1175G), Ap^r	This study
pHK6	Promoter of BT4114 cloned into a *p15 ori* plasmid, Sp^r	This study
pBT4662p	Promoter of BT4662 cloned into a *p15 ori* plasmid, Sp^r	This study

Table S3. Autophosphorylation parameters derived from the cooperative model.

Proteins	Relative efficiency	Phos. efficiency ($min^{-1}mM^{-1}$) [a]	k_{cat} (min^{-1})	K_m (mM)	Cooperativity h	ΔAICc [a]
BT4124-Rec	1	0.16 ± 0.01	1.1 ± 0.04	7.1 ± 0.6	1.6 ± 0.2	-10
BT4124-RR	0.20	0.03 ± 0.01	0.45 ± 0.05	14 ± 4	1.1 ± 0.2	2.1
BT4124-RR^{AG}	0.61	0.10 ± 0.03	0.99 ± 0.14	10 ± 3	1.2 ± 0.3	2.2
BT4124-RR^{AD}	0.97	0.15 ± 0.04	1.1 ± 0.1	7.1 ± 1.5	1.2 ± 0.2	6.1
BT4124-Rec^{AD}	0.97	0.15 ± 0.04	1.4 ± 0.1	9.1 ± 2.2	1.1 ± 0.2	7.5
BT4663-Rec	1	0.07 ± 0.02	0.65 ± 0.1	9.0 ± 2.2	1.6 ± 0.5	1.3
BT4663-RR	0.10	0.007 ± 0.003	0.17 ± 0.03	23 ± 8	1.2 ± 0.2	1.7
BT4663-RR^{AG}	0.31	0.02 ± 0.01	0.26 ± 0.05	12 ± 5	1.3 ± 0.4	2.5
BT4663-RR^{AD}	0.43	0.03 ± 0.01	0.41 ± 0.06	13 ± 4	1.2 ± 0.3	7.0
BT3334-Rec	1	0.29 ± 0.04	1.1 ± 0.1	3.9 ± 0.5	1.5 ± 0.3	-1.5
BT3334-RR	0.23	0.07 ± 0.01	0.49 ± 0.04	7.3 ± 1.4	1.5 ± 0.4	0.1
BT1754-Rec	1	0.06 ± 0.06	1.7 ± 0.7	29 ± 26	0.9 ± 0.3	3.3
BT1754-RR	0.16	0.009 ± 0.007	0.24 ± 0.1	26 ± 18	1.1 ± 0.3	2.1

a. ΔAICc indicates the difference between the corrected Akaike information criterion (AICc) values from the Michaelis-Menten and the cooperative model, $AICc_{coop} - AICc_{MM}$. Negative value of ΔAICc indicates a smaller value of $AICc_{coop}$ than $AICc_{MM}$, suggesting a relative better quality for the cooperative model. Positive value suggests a better quality for the Michaelis-Menten model. AICc is calculated with the following equation:

$$\mathrm{AICc} = N\ln\left(\frac{RSS}{N}\right) + 2K + \frac{2K(K+1)}{N-K-1},$$

in which N is the total number of data points, K is the number of estimated parameters and RSS is the residual sum of squares for the fitting.

Supplemental Text

Kinetic Analyses of Autophosphorylation

As shown below, a simple phosphorylation scheme involves multiple reactions: the binding equilibrium of small-molecule phosphodonor, e.g., phosphoramidate (PAM), the conformational equilibrium to switch RR to the active conformation, autophosphorylation and autodephosphorylation reactions. The exact order or mechanism of the binding and conformational equilibriums is unknown, but can be considered as one overall equilibrium leading to a PAM-bound phosphorylation-competent state. The subsequent autophosphorylation and autodephosphorylation reactions determine the phosphorylation level of RR proteins.

$$\underbrace{\mathrm{RR} + \mathrm{PAM} \overset{K_\mathrm{s}}{\longleftrightarrow} \mathrm{RR{\cdot}PAM} \overset{K_\mathrm{act}}{\longleftrightarrow} \mathrm{RR_A{\cdot}PAM}}_{\text{binding \& conformation equilibriums}} \underbrace{\overset{k_2}{\longrightarrow} \mathrm{RRp}}_{\text{phosphorylation}} \underbrace{\overset{k_{dp}}{\longrightarrow} \mathrm{RR} + \mathrm{pi}}_{\text{dephosphorylation}} \quad (Scheme\ 1)$$

RR autophosphorylation kinetics have been characterized for several proteins, such as CheY, PhoB and NtrX (1-4). In these proteins, phosphorylation causes conformational changes that lead to alteration of the tryptophan fluorescence. Tracking the time course of fluorescence quenching provides sufficient data points for a robust fitting to derive the kinetic parameters. In contrast, HTCS-RRs analyzed here do not contain tryptophan. Phosphorylation reactions were followed by Phos-tag gels with a limited number of time points, which may lead to large uncertainty in parameter estimation, especially coupled with large variations in Phos-tag gel results. Rather than analyzing the entire time course, focusing on the early stage of phosphorylation and fitting initial rates with a simple Michaelis-Menten kinetic model were used for quantitative kinetic analyses.

Full time courses were only used for qualitative assessment of kinetics for a limited set of phosphorylation data at 20 mM PAM. An exponential fit of the fractions of phosphorylated

protein illustrates the reaction progression curve (Fig. 2B, S1B and S2A). The plateau reflects the steady state in which autophosphorylation is balanced by autodephosphorylation. Considering that RR and REC of the same HTCS likely have similar dephosphorylation rates k_{dp}, faster phosphorylation for REC leads to a higher steady-state level of phosphorylation than that of RR. For other PAM concentrations, Phos-tag analyses were usually performed for the first 3-4 time points and 1 later time point (see example in Fig. S2D). Increasing PAM concentrations also result in faster phosphorylation, thus higher steady-state phosphorylation levels (Fig. S2D, 30 min, quantification not shown).

Initial rates obtained from the linear regression of phosphorylation data were fitted with the Michaelis-Menten kinetic equation. Differences in K_m and k_{cat} (Table S1) for HTCS-RRs and HTCS-RECs reflect effects of the inhibitory interaction on different reactions shown in Scheme 1. Decreased K_m values are consistent with an enhanced conformation activation equilibrium (K_{act}) for HTCS-RECs or HTCS-RRs with substitutions in contact residues. Surprisingly, HTCS-RECs and HTCS-RR$^{AG/AD}$ variants also show higher k_{cat} values than HTCS-RRs, suggesting that the phosphorylation catalysis is also affected by interdomain-interactions, leading to higher k_2 values in Scheme 1. The exact mechanism is not clear. It may involve a more complex reaction scheme with additional reactions. For example, dimerization may promote cooperativity in either the catalysis or the conformation activation equilibrium. The limited number of Phos-tag data points is not adequate for a further exploration of the phosphorylation mechanism and the detailed mechanism is not the focus of this study. We chose to derive the k_{cat}/K_m value, a parameter that has been suggested to reflect the energy barrier of the reaction transition state (5), to qualitatively evaluate the inhibitory interdomain interaction for HTCS proteins.

Phosphorylation promotes dimerization of some RRs and a positive cooperativity of autophosphorylation has been observed for some RRs (3, 4). Dimerization can impact the equilibria for PAM binding and conformation activation as well as catalysis. The classic Michaelis-Menten equation may require modification as below to account for cooperativity:

$$\text{Initial rate (Phos. Fraction/time)} = \frac{k_{cat}[PAM]^h}{K_m^h + [PAM]^h}.$$

However, fitting an additional parameter of the cooperativity with limited data points can result in overfitting and large uncertainty associated with the estimated parameters. Table S3 shows the kinetic parameter values obtained from fitting with the cooperative model shown above. Some proteins, such as BT4124-REC, BT4663-REC, BT3334-REC and BT3334-RR, do show a high cooperativity value, suggesting that dimerization of these proteins may play a role in promoting positive cooperativity in autophosphorylation. The corrected Akaike information criterion (AICc) values, a statistical score evaluating the relative quality of models, were computed to compare the cooperative and the Michaelis-Menten model. Lower AICc values, or negative ΔAICc values between the cooperative and the Michaelis-Menten models, reflect better model quality to fit the current dataset. As shown in Table S3, the cooperative model has lower AICc values only for two proteins, BT4124-REC and BT3334-REC. Higher AICc scores for the cooperativity model likely result from the penalty of the additional cooperativity parameter and overfitting with a limited dataset. The Michaelis-Menten model without cooperativity appears to be a better model for most proteins. More data points, especially at the intermediate rate range to define the sigmoidal curve, are needed for a robust fitting using the cooperative model. Nevertheless, the k_{cat}/K_m values from the two models are similar and the conclusion for the inhibitory role of the DBD on autophosphorylation remains valid. For simplicity, data presented in main figures are from kinetic analyses using the Michaelis-Menten model without considering the cooperativity.

All phosphorylation experiments were initially performed without correction for ionic strength changes caused by PAM. As pointed out by a reviewer, addition of PAM could increase the ionic strength, decrease the phosphorylation rate, and impact the kinetic quantification of autophosphorylation (1, 2). We evaluated whether ionic strength has a significant impact on HTCS autophosphorylation. Phosphorylation experiments were performed in parallel, one without ionic strength correction and the other with salt added to keep a constant ionic strength. We chose a constant ionic strength corresponding to 50 mM PAM in the reaction buffer for the convenience of experimental set-up. At pH 7.5, ~14% of ammonium phosphoramidate will be double-charged and 86% will be mono-charged, based on its pK_2 of 8.3 (6), thus the ionic strength will be 1.2 times the PAM concentration. To correct for ionic strength, appropriate volumes of 500 mM PAM were mixed with 600 mM NaCl (1.2x PAM concentration) to reach one tenth of the total reaction volume, and the mixture was added to the reaction to achieve desired PAM concentrations and a constant ionic strength at 0.22 M. As shown in Fig. S1, BT4124 phosphorylation data with or without ionic strength correction displayed nearly identical phosphorylation time courses (Fig. S1A and S1B), phosphorylation rates at 10 mM and 20 mM PAM (Fig. S1C) and kinetic profiles across different PAM concentrations (Fig. S1D). For other HTCS proteins, we focused on the intermediate PAM concentrations of 10 mM and 20 mM where a rate decrease could greatly impact the data fitting. All proteins showed comparable phosphorylation rates for experiments with or without ionic strength correction (Fig. S1E-G). None of the data has a *p* value smaller than 0.05 for comparison of the two ionic strength conditions. Therefore, ionic strength appears not to impact phosphorylation kinetics of HTCS proteins significantly at experimental conditions with PAM below 50 mM. The difference in ionic strength effects for CheY and HTCS proteins may result from differences in the local

protein surface despite the highly conserved active site or differences in phosphorylation detection methods. Quenching of tryptophan fluorescence in CheY depends on the phosphorylation-induced change in the local environment of tryptophan, which may be sensitive to ionic strength. Given no significant effect of ionic strength on phosphorylation, kinetic analyses of HTCSs were performed with all data without ionic strength correction.

1. Da Re SS, Deville-Bonne.D., Tolstykh T, Veron M, Stock JB. 1999. Kinetics of CheY phosphorylation by small molecule phosphodonors. FEBS Lett 457:323-326.
2. Mayover TL, Halkides CJ, Stewart RC. 1999. Kinetic characterization of CheY phosphorylation reactions: comparison of P-CheA and small-molecule phosphodonors. Biochemistry 38:2259-2271.
3. Creager-Allen RL, Silversmith RE, Bourret RB. 2013. A link between dimerization and autophosphorylation of the response regulator PhoB. J Biol Chem 288:21755-69.
4. Fernandez I, Otero LH, Klinke S, Carrica MDC, Goldbaum FA. 2015. Snapshots of conformational changes shed light into the NtrX receiver domain signal transduction mechanism. J Mol Biol 427:3258-3272.
5. Park C. 2022. Visual interpretation of the meaning of k_{cat}/K_M in enzyme kinetics. J Chem Educ 99:2556-2562.
6. Levine D, Wilson IB. 1968. Dipolar ion structure of phosphoramidic acid. Heats of ionization. Inorg Chem 7:818-820.

Sequences of BT-HTCS proteins

>BT4663-RR
MSGDKKRGTILIVEDNNEIRRYLSNGLADLFNTLEAGNGEEALEKLKDNEVDVIVTDVMMPVMDGIKLCK
NVKQNIRT**CH**IPVIILSAKTDIKDQMEGLQMGADDYIPKPFSLAILTTKIQNMMRTRRRMLDKYAKSLEV
EPEKITFNAMDEALLKRAMAIVEKNMDNIEFSTDEFAREMNMSRSNLHLKLKAITGESTIDFIRKIRFNE
AAKLLKDGRYTVAEVSTMVGFNTPSYFATSFKKYFGCLPTEYIKKSKGSGAGGHHHHHHG

>BT4663-Rec
MSGDKKRGTILIVEDNNEIRRYLSNGLADLFNTLEAGNGEEALEKLKDNEVDVIVTDVMMPVMDGIKLCK
NVKQNIRT**CH**IPVIILSAKTDIKDQMEGLQMGADDYIPKPFSLAILTTKIQNMMRTRRRMLDKYAKSGSG
AGGHHHHHHG

>BT4124-RR
MTAPDTLTILVVEDNEELKAFLKNILSENYTVITASNGKEGLQHAVDNIPDLIISDVMMPVMDGLEMIRQ
IKENNNI**CH**IPIIVLSAKASLDDRIAGLEQGIDDYITKPFSATYLKTRIASLLRQRKSLQEIYMAKLTEG
KEIAVAEALTPSQPQITPYDEQFMQKVMEFIEEQMDNAELTIDEFAEHLMLSRTIFYRKLKSIIGLTPVD
FIREVRIKRAAQLIDSGEYNFSQVAYMTGFNDPKYFSKCFKKVVGITPSEYKEKNKGSGAGGHHHHHHG

>BT4124-Rec
MTAPDTLTILVVEDNEELKAFLKNILSENYTVITASNGKEGLQHAVDNIPDLIISDVMMPVMDGLEMIRQ
IKENNNI**CH**IPIIVLSAKASLDDRIAGLEQGIDDYITKPFSATYLKTRIASLLRQRKSLQEIYMAKLTEG
SGAGGHHHHHHG

>BT3334-RR
MRIEDAKMLIVEDNESIKQMLVGIFETFYQVTTASDGVEALDIIQKDMPSIILSDVVMPRMSGTELCKQV
KTDFNT**CH**IPVVLLTARTAVEHNIEGLKIGADDYITKPFNTNLLISRCNNLVNSRRLLQEKFSKQPQAFA
QMLATNPMDKEMLDRAMAIIEQHLDNTDFNVNIFAREMGMARTNLFTKLKAVTGQTPNDFILSIRLKKGA
VMLRNNPELNITEISDRIGFSSSRYFSKCFKEIYHVSPLAYRKGEEKEEEGNEETDQGSGAGGHHHHHHG

>BT3334-Rec
MRIEDAKMLIVEDNESIKQMLVGIFETFYQVTTASDGVEALDIIQKDMPSIILSDVVMPRMSGTELCKQV
KTDFNT**CH**IPVVLLTARTAVEHNIEGLKIGADDYITKPFNTNLLISRCNNLVNSRRLLQEKFSKQPQAGS
GAGGHHHHHHG

>BT1754-RR
MDSSKPSVLIIDDNEDIRSYVHTLLHTDYTVIEAADGSEGIRKAMKYVPDLIISDVMMPGIDGIECCRRL
KSELQT**CH**IPVILLTACSLDEQRIQGYDGGADSYISKPFSSQLLLARVRNLIDSHRRLKQFFGDGQTLAK
EDVCDMDKDFVERFKSLIEEKMGDSGLNVEDLGKDMGLSRVQLYRKIKSLTNYSPNELLRIARLKKAASL
LASSDMTVAEIGYEVGFSSPSYFAKCYKEQFGESPTDFLKRKGSGAGGHHHHHHG

>BT1754-Rec
MDSSKPSVLIIDDNEDIRSYVHTLLHTDYTVIEAADGSEGIRKAMKYVPDLIISDVMMPGIDGIECCRRL
KSELQT**CH**IPVILLTACSLDEQRIQGYDGGADSYISKPFSSQLLLARVRNLIDSHRRLKQFFGDGQSSGA
GGHHHHHHG

DNA fragments used for EMSA

> promoter of BT4114 (for binding of BT4124-RR)

GATGGTAGTGTGGGGTCTCATGCCTCGTCTTTATTCATTTTATGCCGGAAAAACAAAAAATACTATCGTA
AAAACAAATATTACACATCCCACAAGCCCCAAAAACATAATTTTGCACCAGTGAGAACTCTAAGAGTGGA
CACACTCGAATATTAATAATCTAATATAAAAGAACAAAAGATGCCTAAAGGAATGAAGAACGAGACCGAA
ATTCAATAAGGAGG

> promoter of BT4662 (for binding of BT4663-RR)

GATGGTAGTGTGGGGTCTCATGCCTAGGATACAGGAGATAAACAGCTTTTTTAAACAAAAACAACATATG
TCTCAACAAATTTGATATCAAAAAAATCATTTTTGAACAAAGTATGAACAACCTAAATTAAAGAATCTAA
TTAATGAGGTTACATTTGCAACAGTAAAATAACACTTTTAATAATTATAAAACCTAATAAAGTATGAAGA
ACGAGACCGAAATTCAATAAGGAGG

ATG: start codon for the gene transcribed
CAA..CTA: predicted binding sites
